# POSEIDON: A Data Augmentation Tool for Small Object Detection Datasets in Maritime Environments

**DOI:** 10.3390/s23073691

**Published:** 2023-04-02

**Authors:** Pablo Ruiz-Ponce, David Ortiz-Perez, Jose Garcia-Rodriguez, Benjamin Kiefer

**Affiliations:** 1 Department of Computer Technology and Computation, University of Alicante, 03690 San Vicente del Raspeig, Spain; 2 Faculty of Science, University of Tuebingen, 72076 Tuebingen, Germany

**Keywords:** data augmentation, object detection, data imbalance, YOLO, aerial images, maritime environments

## Abstract

Certain fields present significant challenges when attempting to train complex Deep Learning architectures, particularly when the available datasets are limited and imbalanced. Real-time object detection in maritime environments using aerial images is a notable example. Although SeaDronesSee is the most extensive and complete dataset for this task, it suffers from significant class imbalance. To address this issue, we present POSEIDON, a data augmentation tool specifically designed for object detection datasets. Our approach generates new training samples by combining objects and samples from the original training set while utilizing the image metadata to make informed decisions. We evaluate our method using YOLOv5 and YOLOv8 and demonstrate its superiority over other balancing techniques, such as error weighting, by an overall improvement of 2.33% and 4.6%, respectively.

## 1. Introduction

Although object detection has been extensively researched, with a plethora of trained models and architectures available [1], there remain certain areas where large datasets capable of training the most complex deep learning architectures are still lacking. One of these areas pertains to the real-time detection of small vessels, individuals, and other objects in maritime environments using aerial images obtained from drones or small aircraft. Developing robust and precise models for this application would prove to be highly beneficial in search and rescue missions, humanitarian aid efforts, and surveillance [2,3,4] and security operations. However, as we have previously noted in our publication [5], the primary issue is the high cost of capturing such images and the fact that instances in these images tend to be very small. Additionally, maritime environments provide a large factor of variance that has to be dealt with, and variations among the instances to be detected are equally significant.

**Figure 1:**
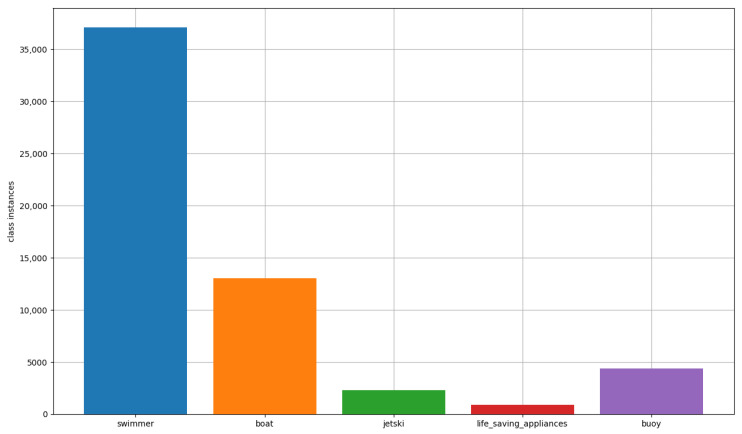
Class instances distribution in the train set of the SeaDronesSee Object Detection v2 dataset.

Our prior research focused on the SeaDronesSee dataset [6], which was introduced in 2021 and encompasses a significant number of aerial images suitable for a variety of applications, including object detection and object tracking. Although additional datasets, such as Seagull [7], are also accessible, SeaDronesSee offers a greater diversity of images with varying attributes, thereby permitting more thorough analysis. Nevertheless, the primary challenge we encountered in our earlier work, and the one we address in this paper, is the significant class imbalance among the various categories present in the dataset, as exemplified in Figure 1.

The motivation of this research is to address the issue of dataset imbalance by examining a range of strategies and proposing a method for generating new samples to reduce the imbalance. Due to the scarcity of available data, the focus has been on utilizing the existing instances in the training set and leveraging image metadata to generate coherent samples that improve object detection for under-represented classes. Initially, we extracted and normalized different objects from the training set. Subsequently, we generated new samples by combining the extracted objects with original images from the dataset. To ensure coherence, we incorporated the extensive metadata available in the dataset to inform our decision-making process.

Finally, we aimed to evaluate the efficacy of our proposed augmentation approach in addressing the issue of class imbalance in the SeaDronesSee dataset. To this end, we compared the performance of two popular object detection architectures, YOLOv5 and the state-of-the-art YOLOv8, trained on the original dataset and the balanced datasets using our augmentation proposal. Both architectures employ class-weighting techniques, similar to those used in our previous research, to mitigate class imbalance. Additionally, all the source code employed for dataset augmentation is available to the public, facilitating the replication of the experiments conducted in this study.

All the work in this research has been focused on the particular case of maritime environments, based on the challenges that we have faced during previous research. However, the method described in the following section could be easily adaptable to any other kind of object detection dataset. In order to adapt this method to other fields, the algorithm in the generation stage might be modified. Different metadata should be used in order to obtain more informed and coherent samples for the new specific field.

The remainder of this paper is structured as follows: Section 2 describes our method, while Section 3 outlines our experimentation and the results obtained. We discuss our findings in Section 4, and outline conclusions and suggestions for future work in Section 5.

## 2. Proposal

This section discusses and analyzes the materials and methods utilized in the study. Furthermore, it examines and analyzes the various ideas that were tested but ultimately not included in the final pipeline, as well as the issues that emerged during the tool’s development and evaluation.

### 2.1. Dataset

**Figure 2:**
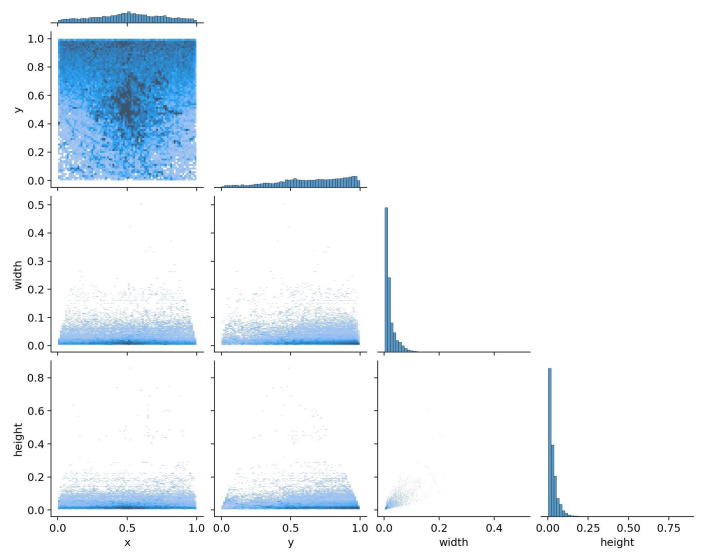
Label correlogram of the different instances in the dataset.

The selected dataset for experimentation was the SeaDronesSee Object Detection v2 [8], which comprises 8931 images and 57,760 instances in the training set. Notably, this dataset is highly imbalanced, as previously observed. Furthermore, the instances are quite small in size, as evidenced in Figure 2. Moreover, the images in the dataset were captured using different cameras, resulting in varying resolutions and aspect ratios. This presented some challenges in the algorithm and is examined in further detail in Section 2.3.

### 2.2. Dealing with Data Imbalance on Object Detection

Imbalanced datasets can negatively affect data-driven algorithms by introducing bias and hindering the understanding of different classes within the data. This can also impact the generalization capabilities of the algorithm. To address this issue, one common solution is to balance the number of instances among different classes by upsampling the minority classes or downsampling the majority classes. In most cases, upsampling is preferred as it typically benefits the generalization capabilities of the algorithm.

Various methods have been developed and tested to balance dataset upsampling for object detection tasks [9,10,11,12,13]. However, additional difficulties arise due to the need to properly modify outputs, such as bounding boxes, when applying transformations to input images. Furthermore, multiple instances of different classes often appear in the same input in most object detection datasets, which cannot be easily addressed by simple augmentation techniques.

In a previous paper, we implemented a technique that did not require downsampling or upsampling. Instead, we assigned weights to each class based on the dataset’s imbalance to provide more importance to less observed classes. This approach is also well-documented [14,15], but adding more instances are generally preferred to increase the variety of observed instances.

Our proposed method generates new inputs by considering the current imbalance of the dataset and leveraging existing instances in the training set. There are other similar methods [10] that propose similar approaches in order to generate new samples. However, as mentioned before, due to the limitations of data availability, the proposed method uses existing instances from the training set. This approach might not provide as much intraclass variance as do other methods, but it will improve results compared to weighting methods. The different stages of the algorithm are thoroughly detailed in the following subsections. The final approaches that are taken and available are discussed as are the additional features that have been implemented, did not end up in the final version, but could guide future lines of work.

### 2.3. Dataset Normalization

**Figure 3:**
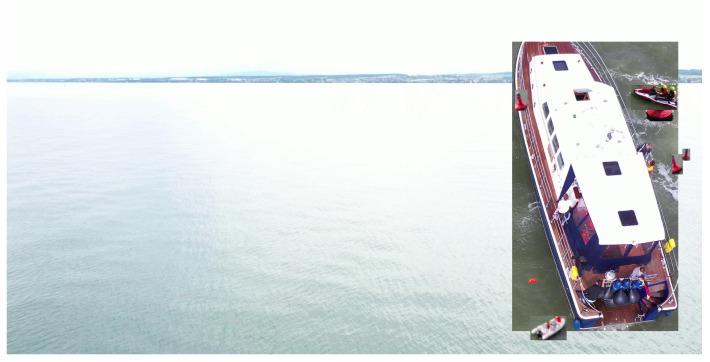
Example of a generated image in which no normalization was applied.

The SeaDronesSee dataset consists of images acquired by various devices at varying resolutions and aspect ratios. Such discrepancies can result in out-of-scale instances when generating images, as depicted in Figure 3. To address this issue, we have implemented a normalization technique, wherein the image with the minimum width across the dataset is selected, and the remaining images are resized in proportion to that width while maintaining their aspect ratios. Additionally, we have adjusted the bounding boxes of all the instances in the resized images to ensure their consistency.

### 2.4. Instance Extraction

**Figure 4:**
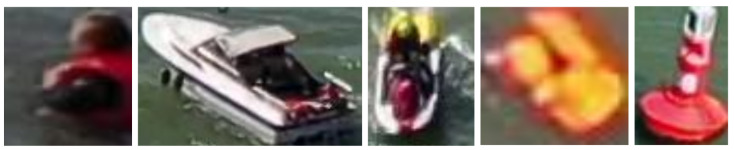
Example instances of the different classes in the dataset.

After the normalization of the dataset, the individual instances in the training set were extracted and classified based on their respective classes. In addition to this, metadata available in the images, such as the camera angle, was considered in order to create more integrated images. Specifically, the camera angle was used to group the instances into 8 categories based on their perspective. Some examples of instances belonging to different classes are shown in Figure 4.

**Figure 5:**
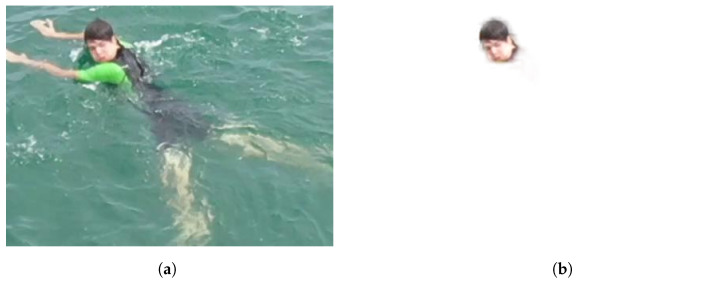
Comparison between the original instance before and after the background removal process. (**a**) Instance with background; (**b**) Effect on the U-2-Net background removal on partially underwater instances.

Since the instances in the dataset lack semantic segmentation information, a portion of the background is visible when extracting the instances. To improve the integration of the instances, we attempted to remove the background using U-2-Net [16] models. However, the results were not satisfactory, as most instances are either partially submerged in water or very small in size. Consequently, the different techniques employed to remove the background produced inconsistent results, as illustrated in Figure 5. Although some of the results were good for larger instances like boats, overall the results were not ideal for an automated pipeline.

In the latest version of our tool, we have made the decision not to remove the background from the extracted images. Nevertheless, we have ensured that the codebase is fully prepared to incorporate this feature once a model or method is developed that produces consistent and satisfactory results in removing the background.

### 2.5. Image Generation

Following the instance extraction process, the generation of new instances is possible using original images from the training set. Each training set image undergoes a process of creating a new one, with instances from other images. Firstly, the majority class instances are ignored to prevent augmentation or continued class imbalance. Secondly, metadata from each image are used to obtain the perspective of the image. A random number of minority class instances, extracted from other images in the training set with the same perspective, are added to the new image. The process is iterated until the class imbalance falls below a specified threshold.

Certain restrictions are applied to increase the realism of the generated samples. For example, as some images contain parts of the sea and the sky, all new instances are placed below the highest instance on the original image to prevent instances from being in the sky. Additionally, since the background from the instances cannot be removed, occlusion between instances is not allowed to avoid unrealistic overlaps. Hence, every instance is placed in a spot without colliding with any other instance.

**Figure 6:**
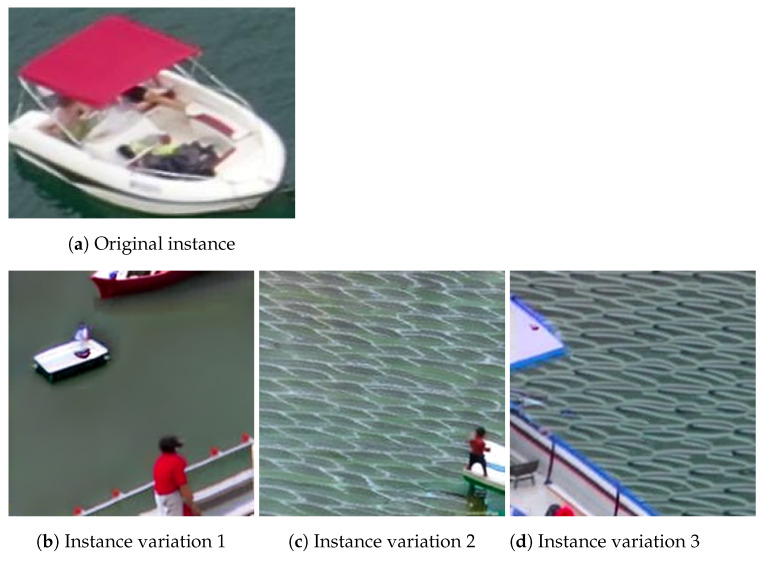
Different image variations using Stable Diffusion and CLIP embeddings.

In addition to the aforementioned steps, an attempt was made to create novel instances through a generative method known as diffusion models [17]. Specifically, we utilized the Image Variations feature of Stable Diffusion [18], which takes as input the CLIP [19] embedding of an image rather than natural language. However, due to the small size of the instances, the generated variations were deemed impractical. An illustration of this process is presented in Figure 6.

**Figure 7:**
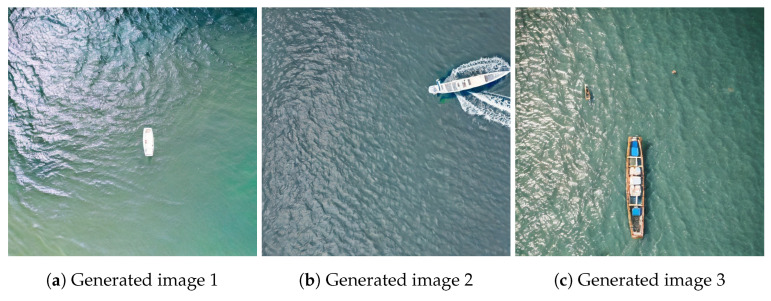
Images generated with Stable Diffusion 2.

Given the lack of success with the image variations method, we further employed the Stable Diffusion 2 technique. This involved using handcrafted prompts for each of the classes, as opposed to the CLIP embedding of the extracted instances. Samples of the resulting images generated through this method can be observed in Figure 7.

Although the second approach seemed to yield superior results, it lacked the desired level of automation. Despite experimenting with various prompts, we were unable to generate different perspectives for the generated instances. Furthermore, the resulting images not only contained the instance, but also a substantial amount of background. As a result, a manual postprocessing step was necessary to crop and annotate the new instances. Given these issues, we ultimately chose not to employ this approach in the final version of the pipeline.

### 2.6. Evaluation

After the generation of a balanced dataset, it is crucial to evaluate the performance of the proposed approach in comparison to other techniques and models.

In our previous work, we utilized YOLOX [20] for object detection. However, in the current study, we have opted for YOLOv5 [21], which is one of the popular object detection models. While the choice of object detector is not a critical aspect, YOLO [22] family detectors have demonstrated strong performance in datasets with similar characteristics [23,24,25]. Furthermore, we chose YOLOv5 because it has proven to be effective in this specific dataset, and it includes an option to balance datasets using weighted errors. Nevertheless, to incorporate more contemporary real-time object detectors, we have also evaluated the performance of the balanced dataset using YOLOv8 [26], which is claimed to be the state-of-the-art (SOTA) for real-time object detection.

To adhere to the principles of our prior work, we utilized a reduced version of the YOLOv5 and YOLOv8 architectures, specifically YOLOv5s and YOLOv8s. In the maritime domain, where images are often captured by drones or small aircraft, the available bandwidth may be insufficient for transmitting images to an external machine or the cloud for real-time predictions. In such scenarios, deploying lightweight models capable of making inferences on edge devices is desirable. The YOLO family has also demonstrated its potential for edge computing applications [27,28].

### 2.7. Final Pipeline

**Figure 8:**
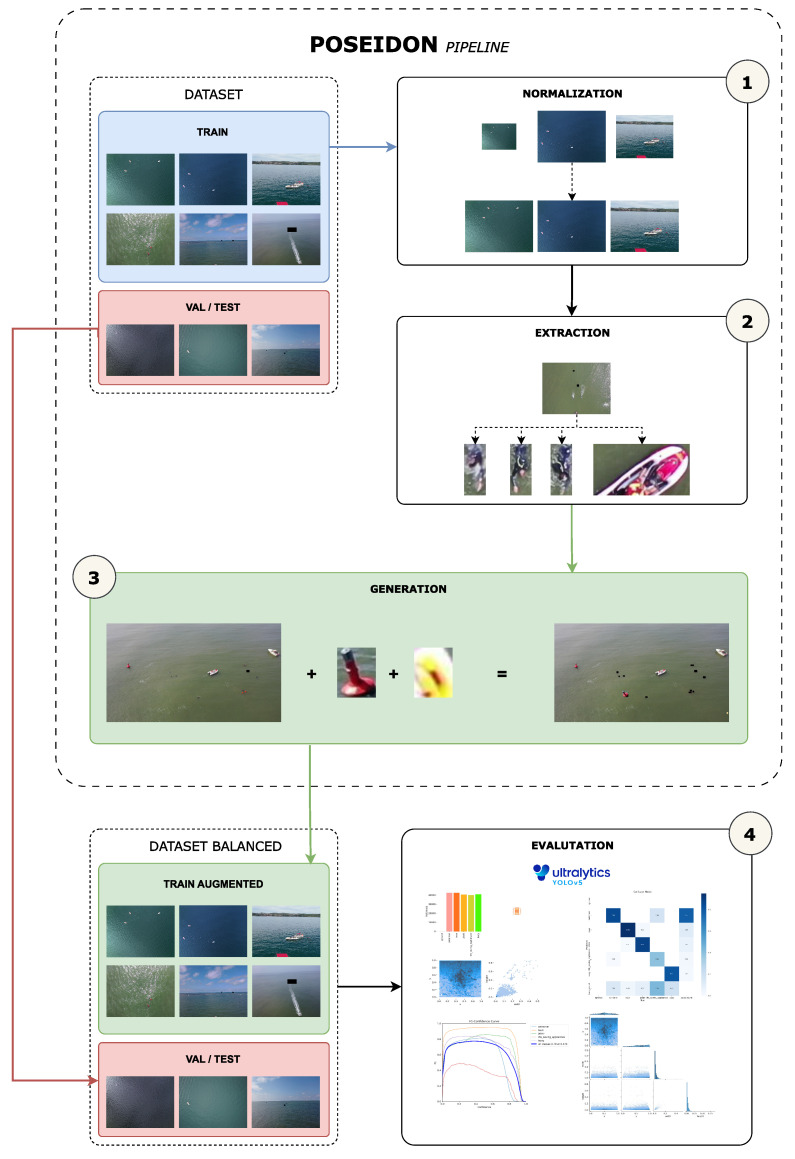
Schema of the final pipeline used.

The different steps we followed to balance the dataset eventually produced the final pipeline, as visualized in Figure 8.

Initially, the training set images of the dataset are subjected to a normalization procedure, as elaborated in Section 2.4. Subsequently, the objects present in the normalized images are extracted using their bounding boxes and grouped using their metadata, as detailed in Section 2.4. Thereafter, in accordance with the description provided in Section 2.5, new samples are produced by utilizing the pre-existing images from the training set and the objects extracted in the previous stage. Finally, the balanced dataset is constructed using the augmented training set and the original validation set and evaluated, as explained in Section 2.6.

## 3. Experimentation and Results

**Figure 9:**
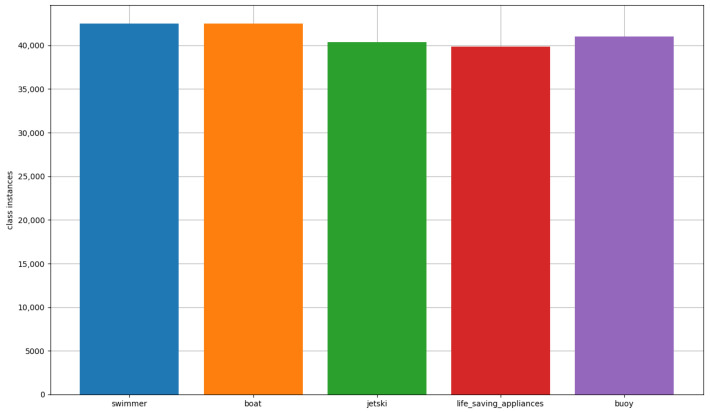
Class instances distribution in the augmented training set after the application of POSEIDON.

After describing the POSEIDON pipeline, we conducted several experiments to evaluate its performance compared to other methods. We utilized the compressed version of the SeaDronesSee Object Detection v2 dataset and trained the model for four iterations on the complete training dataset. The tool was able to balance all classes in the new dataset, resulting in an augmented training set of 27,414 samples, as opposed to the original 8931 images. The distribution of instances among the different classes in the augmented training set is shown in Figure 9.

**Figure 10:**
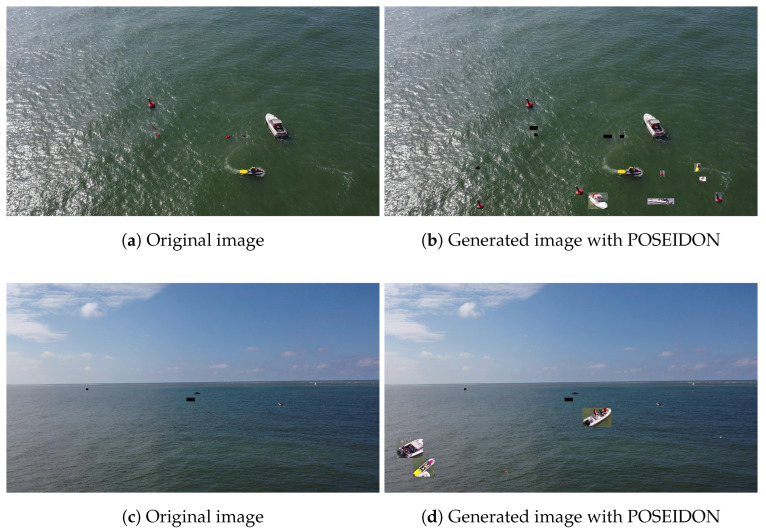
Comparison between the original images and the generated with POSEIDON.

Upon observing the images generated using our proposed method, we can infer that the newly generated images exhibit sufficient coherence. Nonetheless, the lack of proper background removal can result in noticeable seams between the instances and the background. Despite this, a number of the generated images appear to be quite realistic in their appearance. Examples of comparisons between the generated and original images can be seen in Figure 10.

In this study, we trained the proposed augmented dataset in conjunction with the original dataset to evaluate the efficacy of our method. The original training set was balanced using a weighted loss, similar to our prior work. Both datasets were employed to train YOLOv5’s and YOLOv8’s architectures for 60 epochs with a batch size of 16 and input image size of 640 × 640. Default values were utilized for the remaining hyperparameters. We executed the training on an NVIDIA A10 GPU. Further information on the common training parameters employed in our experiments can be found in Table 1.

**Figure 11:**
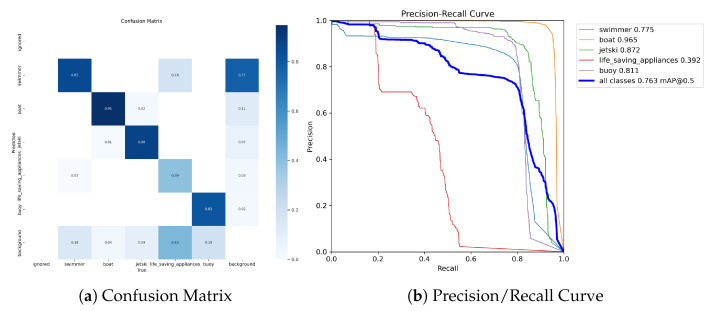
Metrics extracted during the evaluation of the trained model with the augmented dataset.

After analyzing the results presented in Figure 11, we can infer hat the proposed method yields satisfactory outcomes. However, it should be noted that the selected architecture may not possess the full range of capabilities as the original model. Although the use of a larger architecture could potentially yield improved results, it is worth noting that for this specific task, a lightweight model capable of achieving faster inferences was deemed more appropriate, as previously discussed.

## 4. Discussion

Following several experiments, the performance of the dataset augmented with our proposed method can be compared to the predefined baseline. From the outcomes presented in Table 2, the model trained with the augmented dataset using YOLOv5 achieved better mean average precision overall. Notably, the most substantial improvement can be observed in the life-saving appliances category, with a nearly 5.6% boost in performance. This is noteworthy because it was the category with the lowest number of instances in the original training set. Despite the limited amount of training examples, the newly generated images supplied the models with novel illustrations, which improved their ability to accurately identify the objects.

Examination of the other classes shows an average improvement of 2.33% in comparison to the baseline using weighted loss. Although this improvement may not be considered significant, there are several potential areas for further investigation that could lead to more substantial improvements. It is encouraging that the most significant improvements are in the underrepresented classes. As previously noted, there was a nearly 5.6% improvement in the life-saving appliances class and the buoy class, which is the third most underrepresented class, which also experienced a notable improvement.

However, there is one particular class where no improvement was observed. The performance of the Jet Ski instances was almost identical to that of the baseline. Although the level of improvement was proportional to the degree of under-representation of the class in most cases, this was not the case in this instance. Upon analyzing the Jet Ski instances, it was observed that there were always two individuals driving the Jet Ski, which might have led to confusion in the newly generated images, as the small size of the vehicle and the individuals inside might have made it difficult for the models to learn from the generated images.

Looking at the results using the SOTA model, YOLOv8, as delineated in Table 3, it is evident that a comparable pattern of enhancement can be observed. In particular, the YOLOv8 architecture produced superior improvements compared to those generated by YOLOv5, with an overall increase of 4.66%. A clearer correlation between the percentage of improvement and the number of instances can be observed, demonstrating that our proposed approach enhances the models’ ability to detect under-represented classes. Despite the ongoing issues with the Jet Ski class, the results are nevertheless improved. Although YOLOv8 is claimed to be the SOTA and capable of outperforming prior YOLO versions, the overall metrics attained were marginally inferior to those obtained with YOLOv5, as depicted in Table 2. This disparity can be attributed to the fact that we matched the parameters used in YOLOv5 to those employed in YOLOv8, which might not have been optimal. However, since the YOLOv8 research is still in progress, it is challenging to diagnose this minor decline.

The concluding segment of this section compares the predictions generated by the models that were trained using the original dataset versus the augmented dataset using our proposed approach. The images employed for this comparison were the original validation set images from the dataset. Looking at the resultant predictions, it is evident that the model trained with the augmented dataset outperformed its counterpart that was trained with the original dataset.

**Figure 12:**
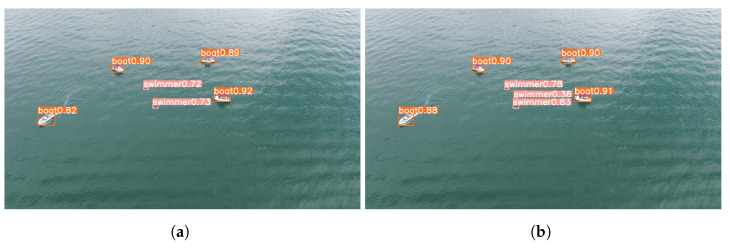
Comparison between the different predictions performed by different models. (**a**) Inference using model trained with the original dataset; (**b**) Inference using model trained with the augmented dataset.

**Figure 13:**
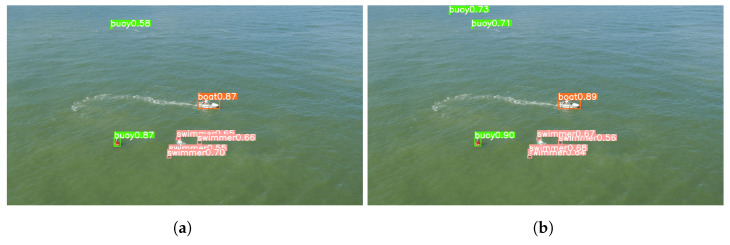
Comparison between the different predictions performed by different models. (**a**) Inference using model trained with the original dataset; (**b**) Inference using model trained with the augmented dataset.

Despite swimmers being the class with the least amount of improvement, Figure 12 demonstrates that the model trained with the augmented dataset is capable of detecting all swimmers, while the model trained with the original dataset is not. Furthermore, as depicted in Figure 13, the former model also exhibits superior performance in detecting small objects that are situated at a greater distance, such as the buoy.

**Figure 14:**
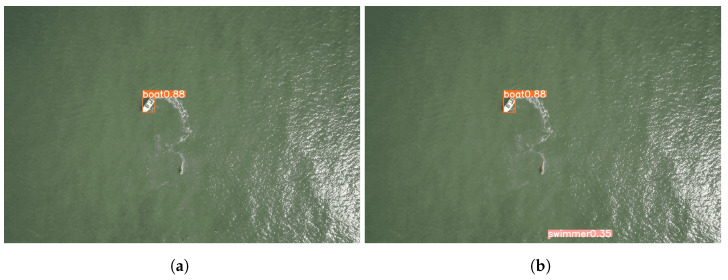
Comparison between the different predictions performed by different models. (**a**) Inference using model trained with the original dataset; (**b**) Inference using model trained with the augmented dataset.

Finally, Figure 14 shows another improved prediction generated by the models trained with the augmented dataset. The figure also illustrates that neither of these models is perfect, as there is still a Jet Ski present in the images that neither model was able to detect. Despite the efficacy of our proposed approach in enhancing performance in highly imbalanced datasets, there remain various avenues for further improvement, as detailed in Section 4.1.

### 4.1. Future Lines of Work

After reviewing the aforementioned results, we notice an enhancement in performance as compared to the proposed baseline. However, previous sections have delved into a thorough analysis of various approaches that did not yield the anticipated outcomes. These approaches, coupled with additional ideas that have been previously introduced, delineate several potential avenues for further research in order to enhance the current methodology.

The removal of the background in the instances could be considered the most significant enhancement that could be implemented to improve the quality and coherence of the generated images. A semantic segmentation labeling of the dataset would be a valuable tool in eliminating the background from the different instances and providing pertinent information for the public dataset.

Within the generation stage of the pipeline, there exist opportunities to increase the variability of the generated images. Firstly, a more sophisticated approach could be taken when determining the size and placement of instances. Presently, our method utilizes only the camera angle to establish perspective and the highest instance to determine the upper limit. However, the SeaDroneSee dataset includes extensive metadata that could be utilized to make more informed decisions, resulting in more realistic image generations. Secondly, the instances extracted from the dataset are replicated identically in the generated images. To introduce greater variance, conventional transformations such as rotation, horizontal flips, and adjustments to contrast and brightness could be applied to the instances during placement.

In order to increase the intraclass variation, the generation of new instances using generative models has been proposed in earlier work. However, this approach has not yet been incorporated into the pipeline for the SeaDronesSee dataset due to the small size of instances and limitations of the state-of-the-art generative models. Nevertheless, with the fast advancements in generative modeling, this approach shows promise for generating new instances based on existing ones, thereby increasing the variance and inducing better generalization in the trained models.

Furthermore, the proposed methodology is not limited to maritime environments and can be applied to object detection datasets in different domains. By making minimal modifications to the codebase, the proposed tool can be employed to balance other datasets. However, certain changes are required in the generation stage, including adjusting instance placement and metadata utilization based on the particular dataset used. It would be beneficial to investigate the performance of these modifications in datasets with larger instances, as some of the approaches that did not work in SeaDronesSee, such as background removal and generative model-based instance generation, may yield better results.

## 5. Conclusions

In this paper, we present a novel method that addresses class imbalance in maritime environment datasets through data augmentation techniques. Our proposed approach has demonstrated its efficacy in improving the performance of object detection models, particularly in scenarios with limited data availability and highly imbalanced datasets. Although the performance gains achieved by our method are modest, they are still significant and demonstrate the potential benefits of addressing class imbalance in this domain. Using YOLOv5, in the most under-represented class, an improvement of 5.6% was achieved, while the overall was 2.3% with respect to the proposed baseline. Additionally, using YOLOv8, the SOTA for real-time object detection, an improvement of 33.3% was achieved in the most under-represented class, while the overall was 4.6% with respect to the proposed baseline.

Based on the results of our experiments, different future lines of work have been defined that would allow for improving the performance obtained with the proposed method. Fortunately, our pipeline is designed in a way that incorporating these improvements into the instance generation process will require no or very simple modifications to the existing codebase.

## Figures and Tables

**Table 1:** Model training configuration.

Parameter	Value
epochs	60
batch_size	16
imgsz	640
optimizer	SGD
label_smoothing	0.0
hyp.lr0	0.01
hyp.lrf	0.01
hyp.momentum	0.937
hyp.weight_decay	0.0005
hyp.warmup_epochs	3.0
hyp.warmup_momentum	0.8
hyp.warmup_bias_lr	0.1

**Table 2:** Comparison of model scores using YOLOv5. Green values indicate an improvement in comparison to the same value of the other model and Red values indicate the contrary. **Bold** represents the overall metric and improvement of a model.

Model	Class	Instances	P	R	mAP50-95	Improvement
Weighted Loss	all	9630	0.874	0.758	**0.429**	**−2.33%**
	Swimmer	6206	0.809	0.789	0.306	−2.29%
	Boat	2214	0.953	0.927	0.675	−2.96%
	Jet Ski	320	0.876	0.872	0.517	+0.38%
	Life-saving appliances	330	0.808	0.420	0.179	−5.58%
	Buoy	560	0.925	0.780	0.469	−3.20%
POSEIDON	all	9630	0.835	0.743	**0.439**	**+2.33%**
	Swimmer	6206	0.814	0.795	0.313	+2.29%
	Boat	2214	0.946	0.947	0.695	+2.96%
	Jet Ski	320	0.815	0.859	0.515	−0.38%
	Life-saving appliances	330	0.689	0.343	0.189	+5.58%
	Buoy	560	0.910	0.773	0.484	+3.20%

**Table 3:** Comparison of model scores using YOLOv8. Green values indicate an improvement in comparison to the same value of the other model and Red values indicate the contrary. **Bold** represents the overall metric and improvement of a model.

Model	Class	Instances	P	R	mAP50-95	Improvement
Weighted Loss	all	9630	0.824	0.633	**0.408**	**−4.66%**
	Swimmer	6206	0.794	0.674	0.291	−0.69%
	Boat	2214	0.915	0.901	0.694	−2.59%
	Jet Ski	320	0.875	0.881	0.562	−2.67%
	Life-saving appliances	330	0.712	0.161	0.105	−33.3%
	Buoy	560	0.823	0.548	0.389	−5.66%
POSEIDON	all	9630	0.828	0.652	**0.427**	**+4.66%**
	Swimmer	6206	0.771	0.688	0.293	+0.69%
	Boat	2214	0.927	0.900	0.712	+2.59%
	Jet Ski	320	0.909	0.900	0.577	+2.67%
	Life-saving appliances	330	0.653	0.205	0.140	+33.3%
	Buoy	560	0.879	0.566	0.411	+5.66%

## Data Availability

The main dataset is available at https://seadronessee.cs.uni-tuebingen.de/ (accessed on 10 February 2023), and the tools and enhanced dataset developed for this work are available at https://github.com/pabloruizp/POSEIDON (accessed on 10 February 2023).
